# Optimal screw positioning in cervical pedicles to avoid complications

**DOI:** 10.55730/1300-0144.5415

**Published:** 2022-03-26

**Authors:** Kerem ATALAR, Zafer Kutay COŞKUN, Ahmet Memduh KAYMAZ

**Affiliations:** 1 Department of Anatomy, Medical Faculty of Gazi University, Ankara, Turkey; 2 Department of Neurosurgery, Medical Faculty of Gazi University, Ankara, Turkey

**Keywords:** Optimal screw positioning, cervical vertebrae, cervical transpedicular screw, 3D reconstruction

## Abstract

**Background/aim:**

Cervical instability can be caused by a variety of factors, including trauma, tumors, or infection. The cervical transpedicular screw (CPS) is one of the most modern procedures for treating cervical instability. Despite the fact that numerous innovative techniques for CPS have been proposed, the appropriate screw entry points and screw directions have yet to be thoroughly established. The aim of this study is to determine the screw insertion angles and screw entry point distances based on reference points, pedicle axis lengths, and pedicle axis intersections for each vertebra from cervical (C) C2 to C7 in both right and left by gender and age groups.

**Materials and methods:**

In this study, computed tomography (CT) images of patients who underwent cervical examination for any reason were evaluated retrospectively. A total of 100 patients (59 men and 41 females), ranging in age from 18 to 79 years (mean 43 years), were randomly selected for the study. Patients with a history of cervical pathology or surgery were excluded. CT images turned into 3D reconstructed images and density settings were made so that bone tissue could be best observed using OsiriX software. Pedicle axis length (PAL), pedicle transverse angle (PTA), pedicle sagittal angle (PSA), distance of screw entry point to lateral notch (DLN), distance of screw entry point to inferior articular process (DIAP), and pedicle axis intersections were measured.

**Results:**

According to our findings, the optimal entry point should be 2–4 mm medial to the lateral notch and 8–12 mm superior to inferior articular process. PTA ranges between 30 to 45°, while PSA ranges between 11 to 15°. Except for the C2 pedicles, which were slightly shorter, the pedicle axis lengths (PAL) were similar from C3 to C7 in the total group. The intersection of the right and left pedicle axes was determined to be the most in C4 (51.21% in females and 72.88% in males).

**Conclusions:**

This study has shown that intersections of the pedicle axis must be considered in both genders, especially in C4. Standardizing optimal entry points and trajectories is crucial for improving the CPS technique’s safety and effectiveness.

## 1. Introduction

The spine consists of 5 parts and is a column of 33–34 vertebrae. Each part shows different morphological features and consists of different numbers of vertebrae. One part, the cervical part, is the most unique and comprises of 7 cervical vertebrae (C1-C7) [1,2]. There are four typical cervical vertebrae (3rd–6th) and three atypical ones (C1, C2, and C7). Despite the fact that cervical vertebrae vary in size, shape, and detail, the main structures of a cervical vertebra can be discussed. Vertebrae are made up of a body, a vertebral arch, and a number of processes. The base of the vertebral arch is formed by two short, thick processes called pedicles. The pedicles extend from the body and join the laminae at the back. The laminae are the flat parts of the vertebral arch that join to form the posterior portion [3].

The neck is a cylindrical structure that houses vital organs and connects the head to the body [4]. The cervical part is the most active part of the vertebral column and contains the body’s most complex joint system. As a consequence of the complexity of this region, it has been determined that 50% of individuals suffer from neck pain at some point in their lives [5]. A better understanding of the region will allow physicians to make accurate diagnoses, which in turn will enable more successful treatment protocols to be identified [4].

Cervical instability can be caused by a number of factors, including trauma, neoplasm, or infection. Stabilization is required for cervical alignment in these situations [6]. For this purpose, cervical transpedicular screw (CPS) is mechanically more powerful than other techniques such as wire technique, interlaminar clamp fixation technique, interfacial screw technique, lower cervical stratification, and lateral mass plate technique because it provides a stronger structure and the risk of failure is lower [7]. CPS technique in subaxial fractures and dislocations of the lower cervical region was first performed by Abumi et al. [8]. Studies have shown that the CPS system provides good support for flexion, extension, torsion, and compression instabilities [9].

Today, one of the most advanced procedures in cervical instability treatment is the CPS technique, and many recent studies have demonstrated the effectiveness of CPS technique in cervical spine surgery [10,11]. The stretch resistance of CPS is 4 times higher than that of the bicortical lateral mass plate technique [9,12]. In addition, thanks to the fact that advanced operating sequence imaging techniques aid surgeons in designating CPS as the fixation technique, the popularity of this technique is increasing [13,14].

Nevertheless, surgical competence and technical knowledge are essential in the execution of the CPS technique, because the surrounding neurovascular structures are susceptible to severe damage in case of a mishap [15–17]. Serious injuries have been reported on the pedicle walls in the operations carried out on experimental models [18, 19] and ones using operational sequence imaging techniques [13].

Some researchers reported damage to the nerve roots caused by superior or inferiorly misplaced screws, damage to the vertebral artery caused by laterally misplaced screws, and damage to the spinal cord and dural sac caused by medially misplaced screws [16, 20–22].

The purpose of this study is to quantitatively evaluate the cervical vertebral pedicle morphology at different cervical vertebral levels and to determine the distance of the screw entry points based on reference points, screw lengths to be used, and screw entrance angles to be set.

## 2. Materials and method

The current study was carried out at Gazi University Medical Faculty Anatomy Department with the approval of the Gazi University Faculty of Medicine Clinical Research Ethics Committee with the decision number 2017-228 dated 08.05.2017. The study involved randomly selected 100 patients (59 males and 41 females) aged 18–79 years (mean 43 years). CT image examinations were reviewed retrospectively. Patients with a pathologic or operative history of the cervical region and patients about whom no quantitative data could be obtained were excluded from the study. CT images were transferred to OsiriX® (Pixmeo, Switzerland) software in DICOM format. With the 3D Volume Rendering feature of the OsiriX software, two-dimensional images were converted into three-dimensional images. Density settings were made so that bone tissue could be best observed. The obtained three-dimensional images were evaluated as transverse surface sections from proximal to distal. For statistical analysis, SPSS 19.0 (IBM corp., New York) software was used.

The conformity of continuous variables to the normal distribution was examined using the Shapiro-Wilk test. Independent sample t-test analysis was used for 2-group comparisons of normally distributed variables. Mann-Whitney U test was used for the comparison of the variables that did not show the normal distribution in 2 groups. The relationship between continuous variables was examined by Spearman correlation analysis. Pearson chi-square test was used for the comparison of categorical variables between independent groups, and McNemar test was used for comparisons between dependent groups. In all statistical analyzes in the study, comparisons with a p value below 0.05 were considered statistically significant.

**Figure 1:**
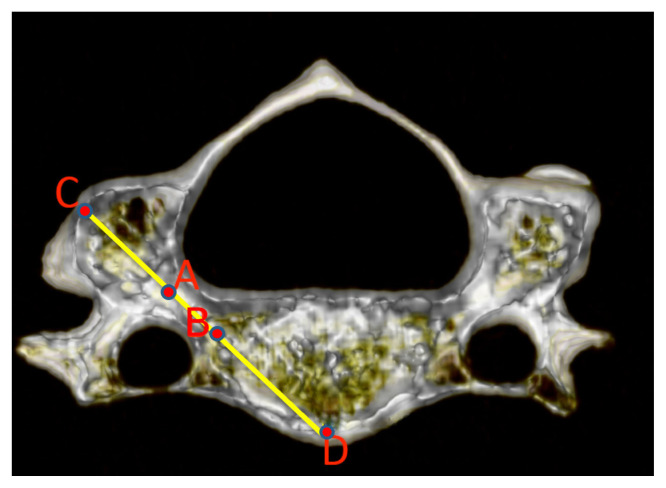
Pedicle Axis. A, middle point of the lateral end of the pedicle (junction with the lateral mass); B, middle point of the medial end of the pedicle (junction with the vertebral body); C, the intersection point of the pedicle axis with the outer wall of the lateral mass; D, the intersection point of the of the pedicle axis with the anterior wall of the vertebral body.

The two ends (junction to lateral mass and vertebral body) of both pedicles (left and right) from C2 to C7 were identified. Anteroposteriorly and laterolaterally, the middle of each cervical pedicle end was identified and marked. The middle of the lateral end (junction with the lateral mass) was designated as point A, and the middle of the medial end (junction with the vertebral body) was designated as point B (Figure 1). Identification of the mid point of the pedicle ending correctly is critical in the identification of the pedicle axis and thus the most appropriate screw transition line.

**Figure 2:**
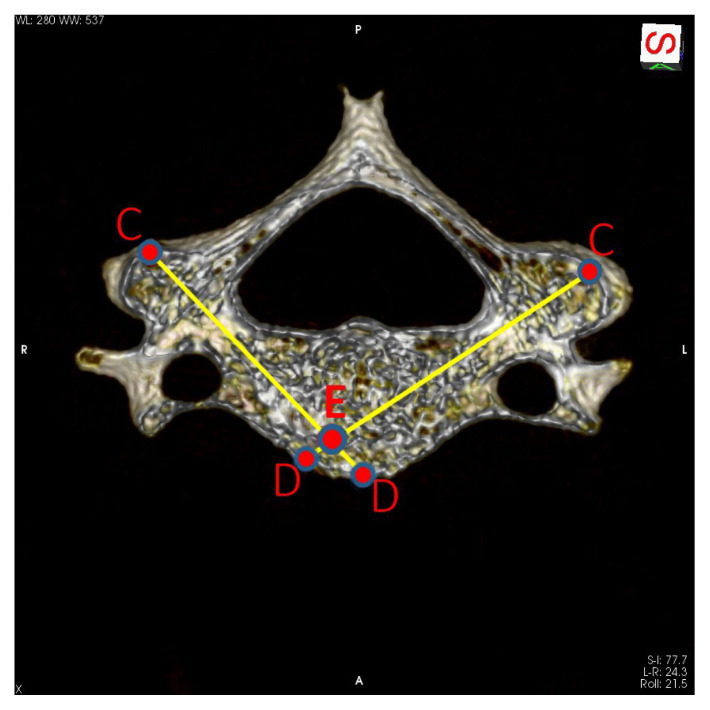
Intersection of the right and left axis. A, middle point of the lateral end of the pedicle (junction with the lateral mass); B, middle point of the medial end of the pedicle (junction with the vertebral body); C, the intersection point of the pedicle axis with the outer wall of the lateral mass; D, the intersection point of the pedicle axis with the anterior wall of the vertebral body; E, intersection of the right and left pedicle axis.

The line connecting points A and B designates the pedicle axis. The intersection point of this line with the outer wall of the lateral mass was marked as point C, and this point designates the screw insertion point. The intersection point of the same line with the anterior wall of the vertebral body was marked as point D, and this point designates the target of the screw. The obtained CD line shows the most suitable screw passage line for the cervical vertebra screw, and the CD line length shows the maximum screw length that can be used (Figure 1). CD line length was appointed as the pedicle axis length (PAL). These procedures were performed separately for the right and left vertebral pedicles. In some of the CT images, it was determined that the right and left pedicle axis crossed the midline (within the vertebral body). The intersection point was designated as point E (Figure 2). This length also represents the maximum screw length that can be used when both the right and left cervical vertebra pedicles are screwed.

**Figure 3:**
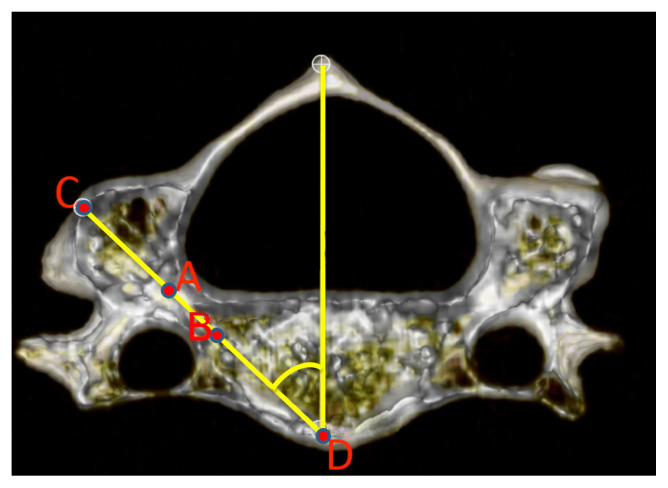
Pedicle transverse angle (PTA). A, middle point of the lateral end of the pedicle (junction with the lateral mass); B, middle point of the medial end of the pedicle (junction with the vertebral body); C, the intersection point of the pedicle axis with the outer wall of the lateral mass; D, the intersection point of the pedicle axis with the anterior wall of the vertebral body.

**Figure 4:**
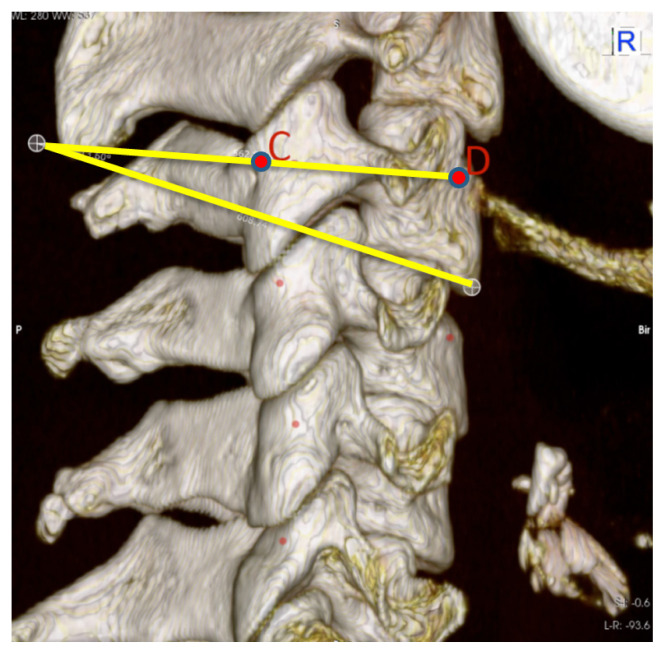
Pedicle sagittal angle (PSA). C, the intersection point of the pedicle axis with the outer wall of the lateral mass; D, the intersection point of the pedicle axis with the anterior wall of the vertebral body.

**Figure 5:**
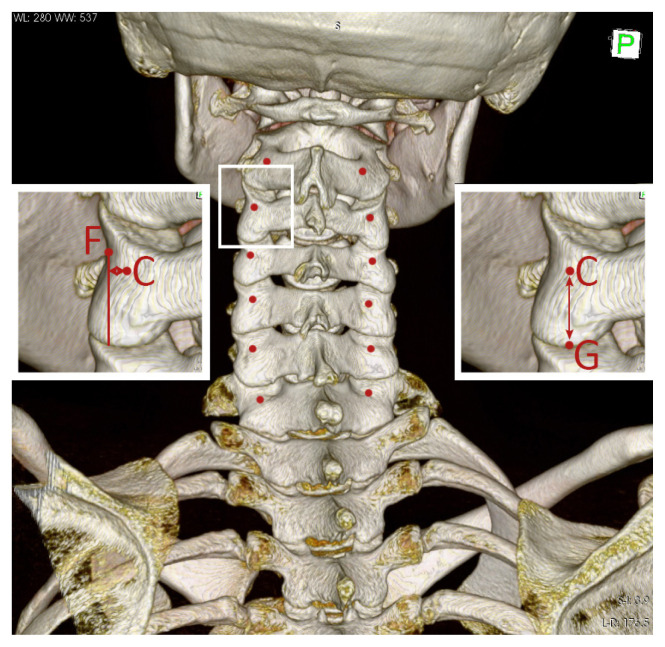
Screw entry point at the lateral mass. C, the intersection point of the pedicle axis with the outer wall of the lateral mass; F, the most medial flank of the lateral mass segment, which connects the superior articular process to the inferior articular process (lateral notch); G, the most inferior border of the inferior articular process.

The pedicle transverse angle (PTA) was defined as the angle formed by the cervical pedicle axis (CD line) and the line drawn parallel to the sagittal plane from point D (Figure 3). The next angle was measured by viewing each vertebra from the lateral side and measuring the angle between the two lines drawn; the first line passing through C and D and the second parallel to the inferior endplate of the vertebral body. The angle between the two lines was appointed as the pedicle sagittal angle (PSA) (Figure 4). On the posterior view, the most medial flank of the lateral mass segment, which connects the superior articular process to the inferior articular process (lateral notch), was designated as point F. A line was drawn vertically, passing through the point F. The perpendicular distance from point C to the line drawn was measured and appointed as the distance to the lateral notch (DLN) (Figure 5). On the same view, the most inferior border of the inferior articular process was appointed as point G. The distance between points C and G was designated as the distance to the inferior articular process (DIAP) (Figure 5).

## 3. Results

For this study, six linear and four angular parameters were measured from 600 vertebrae (C2 to C7 for each patient), representing 1200 pedicles of 100 patients. Measurements of the pedicles from C2 to C7 are given in Table 1. “R” and “L” at the end of the abbreviations indicate right and left. The average values of the PAL (pedicle axis length) were determined 16 mm in C2, 19 mm in C3-C5-C6, 18 mm in C4-C7; PTA (pedicle transverse angle) should be 32 mm in C2, 42 mm in C3, 44 mm in C4-C5, 38 mm in C6, 29 mm in C7; PSA (pedicle sagittal angle) should be 15 mm in C2, 13 mm in C3-C7, 12 mm in C4-C5-C6; DLN (distance to the lateral notch) should be 3 mm in C2-C5, 2 mm in C3-C4, 4 mm in C6-C7; DIAP (distance to the inferior articular process) should be 13 mm in C2, 11 mm in C3, 9 mm in C4, 8 mm in C5-C6-C7.

Only DLNL and DIAPL measurements showed statistically significant differences between males and females (p values 0.018 and 0.008 respectively) in C2. For both measurements, the values of males were statistically significantly higher than those of females. DLNR, DIAPR, and DIAPL showed statistically significant differences between males and females (p values: 0.032, 0.002, 0.007 respectively) in C3. For all three measurements, men’s values were found to be statistically significantly higher than women’s. DIAPR and DIAPL measurements showed statistically significant differences between males and females (p values 0.004 and 0.005, respectively) in C4. For both measurements, men’s values were found to be statistically significantly higher than women’s. In C5, only the DIAPR measurement was statistically significant between males and females (p = 0.048). In this measurement, men’s values were found to be statistically significantly higher than those of women. Measurements of C6 and C7 did not show any statistically significant difference between males and females (p > 0.05).

Except for the C2 pedicles, which were slightly shorter, the pedicle axis lengths (PALR, PALL) were similar from C3 to C7 in the total group. PALR and PALL increased in females from C2 to C3 and decreased from C3 to C7. PALR and PALL in males were similar to the total group. PTAR and PTAL were similar and increased from C2 to C4 and then decreased to C7 in the total group, females and males. PSAR and PSAL were inversely proportional to PTAR and PTAL. PSAR decreased from C2 to C6 and PSAL decreased from C2 to C5 and then increased to C7 in the total group. In females, PSAR and PSAL decreased from C2 to C6, in males decreased from C2 to C5 and then increased to C7 in both genders. DLNR and DLNL both decreased from C2 to C3 and then increased to C7 in group total, females and males. DIAPR decreased from C2 to C7, DIAPL was similar, except C7 was higher than C6. In females, DIAPR and DIAPL both decreased from C2 to C7. In males, DIAPR was similar to females, but DIAPL of C7 was higher than C6.

In Table 2, intersection percentages and the distance between point C and intersection point (CE line length) are given (if an intersection of PALR and PALL is present, this distance represents the maximum screw length that can be used). In females, intersection was most seen in C4 (51.21%) and no intersection was seen in C2 and C7. In males, the most intersection was seen in C4 (25.37%).

The mean values of the age groups are given in Table 3. A significant difference (p < 0.005) between age groups was found in C2, C3, and C7. Accordingly, DLNR and DLNL in C2, DLNL in C3 were increasing; DIAPR in C2; PSAL in C3; and PTAL parameters in C7 were decreasing with age. No other correlation between age groups was found.

## 3. Discussion

Compared to other parts of the vertebral column, the cervical part has the most unique features and contains the most complex joint system of the body [4]. As a result of this complexity, 50% of individuals complain of neck pain at some point in their lifetime [5]. The most frequently injured part of the vertebral column is the cervical part, with a rate of 55% [23]. In patients with cervical vertebral injury, a fracture of 0.9%–2% was detected [24,25]. Interlaminar clamp fixation technique, interfacet screw technique, lower cervical wiring, and lateral mass screwing technique can be used for treatment purposes. Although these treatment approaches are effective in cervical stabilization, mechanically, the cervical transpedicular screwing technique provides a stronger structure than other techniques and is less likely to fail [9,26]. Biomechanical studies have reported that cervical pedicle screws provide superior stabilization to other posterior cervical fixation applications [26–28]. Cervical pedicle screws are not only effective for traumatic or nontraumatic conditions, but also for the treatment of diseases such as kyphosis and spondyloarthropathy [29–31].

This technique improves the bone union rate by providing stabilization of pedicle screws in slow bone union requiring high biomechanical immobility and can help with rehabilitation by shortening the time after surgery [32]. Animal studies and cadaveric trials have shown that the CPS technique provides stronger stabilization, fixation, and pull-out resistance compared to lateral mass screws [9, 33, 34]. In the studies performed, the pull-out resistance of the cervical pedicle screw was between 1214 Newton (N) and 332 N [35] and between 677 N and 355 N [36].

Under increasing cyclic loads, it was observed that pedicle screws failed due to pedicle fracture rather than being dislocated; in the lateral mass screws, it was observed that the screw was loosened out due to poor fixation [35]. According to a study performed in 2012, cervical pedicle screw application can be used not only in adult individuals but also in children [37]. In addition, there are studies reporting that cervical transpedicular screwing is possible in C2 even in children aged 2 to 10 years [38]. For these reasons, surgeons’ interest in cervical pedicle screws is rapidly increasing [30, 32, 39].

However, the cervical transpedicular screwing technique is a procedure that requires surgical competence and technical knowledge, since serious damage to the surrounding neurovascular structures may occur [15–17, 20]. In experimental models [18,19] and interventions using operation sequence imaging techniques, serious injuries to pedicle walls have been reported. Schmidt et al. (2010) [40] compared the lateral mass screwing technique to the cervical transpedicular screwing technique and reported that the biomechanical lateral mass screwing technique provides adequate stabilization. Therefore, the technically demanding cervical screwing technique should be avoided [31]. However, in a study, with 89.7% of well-positioned screws, CT-navigated pedicle screws in the subaxial cervical spine showed great accuracy [11]. Installation of pedicle screws in cervical vertebrae is more difficult than in thoracic or lumbar vertebra due to the smaller pedicle sizes, individual differences in pedicle anatomy, and poor outcomes of complications in this area [15–17, 20, 26, 33, 41].

It is reported that there is significant heterogeneity in the reporting of landmarks for the appropriate CPS technique across studies [10]. Many studies have suggested the use of topographic markers [42], precise measurements of the parameters [40, 43], and the use of advanced surgical techniques or devices [44–46] to increase the precision of pedicle screw placement. Morphological examinations of the cervical pedicles, whether by direct or CT measurement, are of great importance to prevent complications in operations using this method [22]. Determining the ideal pedicle trajectory is crucial to measuring the pedicle axis properly. The ideal pedicle trajectory must pass through the center of the pedicles in all 3 planes [47]. We ensured that by determining the center on both pedicle ends.

The cervical transpedicular screwing technique was first described by Abumi et al. (1994) [8] in 1994, and the use of the technique has grown steadily [48]. Several researchers have conducted studies to improve the technique of cervical transpedicular screwing [37, 49–51]. Abumi et al. (1994) [8] stated that the screw entry point should be slightly lateral to the mid-point of the lateral mass and close to the lower border of the superior articular facet.

To determine the ideal screw entry point, researchers have identified various reference points [19, 45, 47, 52, 53]. Ebraheim et al. (1997) [52] used the vertical line combining the outer edges of the lateral mass and a horizontal line passing through the inferior edge of the superior articular facet; Ludwig et al. (2000) [54] used the vertical plane of the inferior edge of the superior articular facet and the medial edge of the superior articular facet; Rao et al. (2008)[53] used the lateral edge of the lateral mass and the lower edge of the superior articular facet; Lee et al. (2011) [19] used the lateral notch; Herrero et al. (2016) [47] used the contribution point of the spinous process with the lamina of vertebral arch as the reference point. The reference points of Karaikovic et al. (2000) [45] are the same points that we use in our study. We preferred the lower edge of the inferior articular process as one of the reference points instead of the superior articular process, to prevent mismeasurements caused by covering the top of the superior articular process. Compared to Karaikovic et al. (2000) [45] our DLN findings are similar in C2 to C6 in both genders and in C7 in males. In females, our findings in C7 were higher. Our IAPD findings were similar to Karaikovic et al. (2000) in C2 to C4 and lower in C5 to C7 in both genders.

There are many studies on PAL measurement [47, 53, 55–58]. Compared to our results, PAL was measured higher by Herrero et al. (2016)[47], Rao et al. (2008) [53], Sakamoto et al. (2004) [55], Wasinpongwanich et al. (2014) [57] and Westermann et al. (2018) [58], and lower by Eldin (2014) [56]. Herrero et al. (2016) [47], Rao et al. (2008) [53], Sakamoto et al. (2004) [55], Chazono et al. (2006) [59] and Wasinpongwanich et al. (2014) [57] performed PTA measurements and compared to us they found higher values while Eldin (2014) [56] found lower, Karaikovic et al. (1997) [60] and Ludwig (2000) [54] found similar results. PSA results measured by Rao et al. (2008) [53], Karaikovic et al. (1997) [60] and Wasinpongwanich et al. (2014) [57] were significantly lower than ours.

The pedicle axis intersection parameter has not been seen in any of the prior studies. If intersection is present, pedicle screws must be selected based on this factor.

While some of our findings show similar results to studies prior to ours, others are inconsistent with them. The lack of consistency can be mainly due to age differences between the individuals, ethnic groups, gender distribution, different softwares used for measurements, different measurement techniques, and the difference in materials used (CT/MR).

The main limitation of this study is absence of a clinical assessment of the CPS technique.

## 4. Conclusion

According to our findings, the average values of the PAL (pedicle axis length) should be 16 mm in C2, 19 mm in C3-C5-C6, 18 mm in C4-C7; PTA (pedicle transverse angle) should be 32 mm in C2, 42 mm in C3, 44 mm in C4-C5, 38 mm in C6, 29 mm in C7; PSA (pedicle sagittal angle) should be 15 mm in C2, 13 mm in C3-C7, 12 mm in C4-C5-C6; DLN (distance to the lateral notch) should be 3 mm in C2-C5, 2 mm in C3-C4, 4 mm in C6-C7; DIAP (distance to the inferior articular process) should be 13 mm in C2, 11 mm in C3, 9 mm in C4, 8 mm in C5-C6-C7. Our findings demonstrate that pedicle axis intersections must be considered in both genders, particularly in C4. Standardizing optimal entry points and trajectories is critical for improving the safety and effectiveness of the CPS technique. We believe that our findings support previous anatomical studies in this field.

## Figures and Tables

**Table 1:** Comparison of parameters for men and women for each vertebra (C2 to C7). Right pedicle axis length, PALR; left pedicle axis length, PALL; right pedicle transverse angle, PTAR; left pedicle transverse angle, PTAL; right pedicle sagittal angle, PSAR; left pedicle sagittal angle, PSAL; distance to the lateral notch on the right side, DLNR; distance to the lateral notch on the left side, DLNL; distance to the inferior articular process on the right side, DIAPR; distance to the inferior articular process on the left side, DIAPL.

Vertebra	Parameter (mm)	Female (N = 41)	Male (N = 59)	Total (N = 100)	p
**C2**	PALR	17.00 (9.20–26.70)	15.40 (10.20–37.3)	16.00 (9.20–37.30)	0.844#
	PALL	18.80 (8.30–28.30)	16.30 (10.6–27.7)	16.55 (8.30–28.30)	0.614#
	PTAR	31.47 ± 6.75	30.7 ± 7.05	30.90 ± 6.90	0.570*
	PTAL	36.06 ± 7.87	33.3 ± 7.62	34.43 ± 7.81	0.080*
	PSAR	15.52 (6.70–46.40)	13.64 (4.70–24.50)	14.49 (4.70–46.44)	0.068#
	PSAL	17.0 ± 4.61	15.3 ± 4.93	15.90 ± 4.84	0.097*
	DLNR	2.27 (1.00–9.70)	2.79 (1.15–6.70)	2.57 (1.00–9.70)	0.144#
	DLNL	2.33 (0.80–4.32)	2.71 (0.77–7.10)	2.50 (0.77–7.10)	0.018#
	DIAPR	11.5 ± 2.56	12.6 ± 2.98	12.86 ± 12.05	0.051*
	DIAPL	11.20 (7.20–17.70)	13.10 (5.80– 28.90)	12.10 (5.80–28.90)	0.008#
**C3**	PALR	25.40 (13.40– 32.50)	17.80 (13.80– 46.10)	18.90 (13.40–46.10)	0.695#
	PALL	26.20 (12.60– 33.80)	17.60 (13.0– 47.60)	18.55 (13.0–47.60)	0.760#
	PTAR	42.00 ± 4.15	40.80 ± 3.62	41.29 ± 3.87	0.137*
	PTAL	42.87 ± 4.31	43.00 ± 5.18	42.94 ± 4.82	0.909*
	PSAR	13.36 (5.80–30.47)	13.43 (2.86– 22.89)	13.39 (2.86–30.47)	0.925#
	PSAL	13.98 (7.90–28.97)	13.70 (4.30– 23.22)	13.80 (4.30–28.97)	0.544#
	DLNR	1.84 (0.60–15.70)	2.38 (0.70–7.20)	2.22 (0.60–15.70)	0.032#
	DLNL	2.23 (0.60–4.91)	2.36 (0.68–7.10)	2.30 (0.60–7.10)	0.277#
	DIAPR	10.03 (1.10–15.80)	11.40 (4.50–24.00)	10.80 (1.10–24.00)	0.002#
	DIAPL	10.60 (5.70–15.30)	11.45 (6.20–21.60)	11.20 (5.70–21.60)	0.007#
**C4**	PALR	25.70 (13.30– 30.90)	17.80 (11–46.10)	18.55 (11–46.10)	0.470*
	PALL	27.00 (13.00– 34.30)	17.12 (12.40–50.90)	18.90 (12.40– 50.90)	0.913*
	PTAR	44.90 ± 3.90	44.83 ± 5.18	44.86 ± 4.68	0.941#
	PTAL	44.72 ± 4.36	46.55 ± 4.75	45.80 ± 4.66	0.530#
	PSAR	12.05 ± 3.16	12.07 ± 3.73	12.06 ± 3.48	0.978#
	PSAL	12.62 ± 4.02	12.46 ± 4.05	12.53 ± 4.02	0.854#
	DLNR	2.46 (1.00–5.90)	2.69 (0.95–6.30)	2.62 (0.95–6.30)	0.305*
	DLNL	2.40 (1.08–4.21)	2.68 (0.63–5.70)	2.59 (0.63–5.70)	0.202*
	DIAPR	8.90 (1.64–12.30)	10.30 (4.29– 18.60)	9.20 (1.64–18.60)	**0.004** *
	DIAPL	8.81 ± 2.36	10.11 ± 2.16	9.57 ± 2.33	**0.005** #
**C5**	PALR	25.20 (13.60–31.60)	18.90 (14.10– 41.50)	20.30 (13.60–41.50)	0.666#
	PALL	25.20 (12.60– 32.60)	17.60 (12.90– 44.60)	18.30 (12.60–44.60)	0.847#
	PTAR	42.06 (25.60– 54.68)	44.62 (22.60– 58.23)	43.65 (22.60–58.23)	0.305#
	PTAL	44.42 ± 5.31	44.45 ± 5.72	44.44 ± 5.53	0.978*
	PSAR	11.91 ± 3.24	11.81 ± 3.34	11.85 ± 3.28	0.879*
	PSAL	11.94 (6.40–20.70)	12.45 (5.50– 27.98)	12.33 (5.50–27.98)	0.406#
	DLNR	3.17 ± 1.11	3.51 ± 1.31	3.37 ± 1.24	0.176*
	DLNL	3.10 (1.08–6.90)	2.94 (0.40–8.60)	3.04 (0.40–8.60)	0.801#
	DIAPR	7.80 (1.92–12.90)	8.70 (3.40–40.60)	8.40 (1.92–40.60)	**0.048** #
	DIAPL	8.40 (3.63–11.70)	8.80 (3.11–14.80)	8.70 (3.11–14.80)	0.091#
**C6**	PALR	22.40 (14.70–31.80)	19.10 (12.70– 41.60)	19.50 (12.70–41.60)	0.391#
	PALL	24.60 (12.50– 31.80)	18.20 (12.60– 40.90)	18.90 (12.50–40.90)	0.944#
	PTAR	36.72 ± 5.98	38.13 ± 6.85	37.55 ± 6.51	0.291#
	PTAL	38.31 ± 4.75	39.46 ± 6.70	38.99 ± 5.98	0.344*
	PSAR	10.50 (5.60–31.74)	12.43 (4.90– 22.85)	11.48 (4.90–31.74)	0.076*
	PSAL	11.89 (4.70–19.40)	13.08 (5.97– 27.40)	12.82 (4.70–27.40)	0.305#
	DLNR	3.67 (1.70–20.30)	4.07 (1.73–10.40)	3.92 (1.70–20.30)	0.150*
	DLNL	3.68 ± 1.07	3.89 ± 1.52	3.80 ± 1.35	0.434#
	DIAPR	7.66 ± 2.07	8.43 ± 2.66	8.12 ± 2.45	0.121#
	DIAPL	8.39 ± 2.14	8.48 ± 2.61	8.44 ± 2.42	0.862#
**C7**	PALR	19.90 (11.70– 44.90)	17.00 (12.50– 42.60)	18.50 (11.70–44.90)	0.889#
	PALL	22.60 (11.70– 44.80)	17.40 (11.10– 42.30)	18.80 (11.10–44.80)	0.997#
	PTAR	27.40 (19.20– 48.00)	28.57 (21.05– 47.70)	28.31 (19.20–48.00)	0.379#
	PTAL	30.10 (14.70– 45.00)	29.37 (21.22– 51.75)	29.94 (14.70–51.75)	0.739#
	PSAR	13.10 (4.94–22.92)	13.49 (16.00– 25.98)	13.16 (16.00–25.98)	0.239#
	PSAL	13.68 ± 4.29	14.68 ± 4.27	14.27 ± 4.28	0.253*
	DLNR	4.20 (1.61–7.40)	4.98 (1.67–12.30)	4.51 (1.61–12.30)	0.074#
	DLNL	4.56 (2.60–7.20)	4.91 (2.16 –15.20)	4.80 (2.16–15.20)	0.416#
	DIAPR	7.30 (3.20–13.00)	8.40 (3.70 –22.40)	8.10 (3.20–22.40)	0.481#
	DIAPL	8.00 (2.54–13.50)	8.90 (2.47–23.60)	8.70 (2.47–23.60)	0.801#

* Independent sample t test;

# Mann Whitney U test

**Table 2:** Intersection percentages of rigt and left pedicle axis in men and women. The distance between point C and intersection point (CE line length).

Right		Female (N = 41)		Male (N = 59)	
		Left	Right	Left	
**C2**	**N**	-	-	3 (5.08%)	3 (5.08%)
	**Mean (mm)**	-	-	25.80 ± 1.34	25.46 ± 0.47
**C3**	**N**	15 (36.58%)	15 (36.58%)	30 (50.84%)	30 (50.84%)
	**Mean (mm)**	27.43 ± 1.43	27.58 ± 1.87	29.95 ± 3.35	30.97 ± 3.19
**C4**	**N**	21 (51.21%)	21 (51.21%)	43 (72.88%)	43 (72.88%)
	**Mean (mm)**	26.99 ± 1.59	27.70 ± 1.95	29.73 ± 2.85	30.20 ± 3.11
**C5**	**N**	12 (29.26%)	12 (29.26%)	32 (54.23%)	32 (54.23%)
	**Mean (mm)**	28.39 ± 1.91	28.45 ± 1.51	31.53 ± 3.26	31.42 ± 2.20
**C6**	**N**	3 (7.31%)	3 (7.31%)	15 (25.42%)	15 (25.42%)
	**Mean (mm)**	29.86 ± 1.28	30.56 ± 0.83	31.91 ± 2.89	32.98 ± 2.26
**C7**	**N**	-	-	2 (3.38%)	2 (3.38%)
	**Mean (mm)**	-	-	36.80 ± 3.12	38.10 ± 1.83

**Table 3:** Mean values and the relation of the age groups. Right pedicle axis length, PALR; left pedicle axis length, PALL; right pedicle transverse angle, PTAR; left pedicle transverse angle, PTAL; right pedicle sagittal angle, PSAR; left pedicle sagittal angle, PSAL; distance to the lateral notch on the right side, DLNR; distance to the lateral notch on the left side, DLNL; distance to the inferior articular process on the right side, DIAPR; distance to the inferior articular process on the left side, DIAPL.

Vertebra	Parameter (mm)	18–30 (N = 25)	31–50 (N = 41)	51+ (N = 34)	p
**C2**	PALR	17.38 (10.80– 28.40)	16.6 (9.20–27.10)	17.8 (10.20–37.30)	0.633#
	PALL	17.76 (11.70– 27.70)	17.16 (8.30–28.30)	17.52 (10.60– 27.60)	0.896#
	PTAR	31.33 ± 7.21	32.14 ± 5.95	29.37 ± 7.59	0.142*
	PTAL	34.08 ± 7.94	36.29 ± 8.19	32.41 ± 6.84	0.132*
	PSAR	15.22 (4.70–21.24)	15.21 (6.70–27.85)	14.13 (5.71–46.44)	0.075#
	PSAL	16.59 ± 5.03	16.97 ± 4.81	14.35 ± 4.42	0.027*
	DLNR	2.34 (1.00–6.90)	2.81 (1.15–9.70)	3.49 (1.22–5.80)	**0.002** #
	DLNL	2.39 (1.23–4.30)	2.63 (0.77–7.10)	3.30 (1.35–6.10)	**0.003** #
	DIAPR	13.25 ± 1.86	12.21 ± 2.55	11.26 ± 3.50	**0.004** *
	DIAPL	12.71 (8.70–18.10)	12.09 (5.80–17.90)	11.86 (7.60–28.90)	0.188#
**C3**	PALR	22.92 (14.20– 32.80)	22.56 (13.40– 38.10)	22.67 (13.80– 46.10)	0.960#
	PALL	23.18 (14.40– 36.30)	22.86 (13.00– 39.20)	22.22 (1.30–47.60)	0.923#
	PTAR	40.85 ± 3.19	41.27 ± 3.91	41.62 ± 4.33	0.706*
	PTAL	43.19 ± 4.21	44.00 ± 4.90	41.47 ± 4.90	0.177*
	PSAR	14.20 (7.40–21.60)	13.8 (5.68–30.47)	11.86 (2.86–23.32)	0.092#
	PSAL	15.16 (7.10–23.22)	14.81 (5.25–28.97)	12.59 (4.30–22.70)	**0.044** #
	DLNR	2.92 (1.20–15.70)	2.32 (0.60 – 5.00)	2.78 (0.70–7.20)	0.342#
	DLNL	2.21 (0.60–3.73)	2.18 (0.84–3.93)	3.01 (0.68–7.10)	**0.005** #
	DIAPR	10.32 (6.50–12.20)	10.60 (5.90–15.80)	10.98 (1.10–24.00)	0.526#
	DIAPL	10.48 (5.70–14.20)	10.55 (6.20–15.30)	11.46 (6.30–21.60)	0.231#
**C4**	PALR	22.71 (14.40– 34.00)	22.34 (11.00– 37.40)	22.79 (12.80– 46.10)	0.875#
	PALL	22.7 (13.50–36.30)	22.4 (13.00–38.20)	22.97 (12.40– 50.90)	0.973#
	PTAR	44.27 ± 3.47	45.38 ± 4.92	44.66 ±5.18	0.623*
	PTAL	45.33 ± 4.37	46.79 ± 4.96	44.94 ± 4.37	0.200*
	PSAR	12.37 ± 2.39	12.43 ± 3.29	11.37 ± 4.27	0.329*
	PSAL	12.39 ± 3.76	13.19 ± 3.67	11.80 ± 4.55	0.285*
	DLNR	2.67 (1.06–4.80)	2.86 (1.16–6.30)	2.91 (0.95–6.00)	0.881#
	DLNL	2.87 (1.08–5.40)	2.52 (1.11–5.70)	2.70 (0.63–5.50)	0.505#
	DIAPR	9.68 (5.40–13.00)	9.77 (1.64–15.10)	9.70 (5.50–18.60)	0.736#
	DIAPL	10.02 ± 2.14	9.46 ± 2.32	9.35 ± 2.47	0.636*
**C5**	PALR	22.30 (13.70– 33.70)	22.49 (13.60– 38.30)	23.61 (14.60–41.50)	0.592#
	PALL	22.98 (14.10– 33.50)	22.17 (12.60– 38.80)	23.42 (12.90–44.60)	0.651#
	PTAR	43.36 (25.60– 54.78)	43.17 (33.77– 58.23)	42.41 (22.60 – 51.94)	0.725#
	PTAL	44.52 ± 5.42	45.16 ± 6.12	43.5 ± 4.82	0.565*
	PSAR	11.84 ± 3.31	12.23 ± 3.12	11.4 ± 3.48	0.490*
	PSAL	12.37 (5.50–22.04)	12.96 (8.31–27.98)	12.53 (5.-7–22.76)	0.808#
	DLNR	3.07 ± 1.05	3.54 ± 1.34	3.37 ± 1.21	0.336*
	DLNL	3.03 (1.49–5.50)	3.36 (1.27–7.30)	3.01 (0.40–8.60)	0.269#
	DIAPR	8.71 (3.75–14.30)	9.39 (1.92–40.60)	8.28 (3.40–12.40)	0.816#
	DIAPL	8.94 (3.63–14.40)	8.67 (4.40–14.80)	8.21 (3.11–12.60)	0.649#
**C6**	PALR	22.92 (14.70– 33.20)	21.74 (12.70– 35.70)	23.49 (14.20–41.60)	0.394#
	PALL	22.59 (13.80– 35.90)	21.99 (12.50– 38.80)	22.77 (13.10–40.90)	0.924#
	PTAR	38.50 ± 6.48	37.38 ± 7.04	37.12 ± 5.96	0.691*
	PTAL	39.28 ± 5.37	39.85 ± 6.57	37.73 ± 5.58	0.450*
	PSAR	12.28 (4.90–18.50)	12.81 (5.17–31.74)	11.61 (5.57–22.85)	0.479#
	PSAL	13.01 (4.70–19.28)	12.73 (6.16–22.38)	12.70 (5.97–27.40)	0.708#
	DLNR	3.61 (1.70–7.20)	4.32 (2.17–10.40)	4.57 (1.98–20.30)	0.112#
	DLNL	3.61 ± 1.30	4.16 ± 1.32	3.49 ± 1.36	0.071*
	DIAPR	8.65 ± 2.20	8.08 ± 2.49	7.75 ± 2.57	0.351*
	DIAPL	8.72 ± 2.07	8.41 ± 2.18	8.26 ± 2.93	0.615*
**C7**	PALR	20.91 (12.90– 31.80)	19.44 (11.70– 37.50)	21.56 (12.20–44.90)	0.461#
	PALL	21.85 (11.70– 33.10)	19.60 (11.30– 35.70)	21.19 (11.10–44.80)	0.362#
	PTAR	30.41 (19.20– 40.53)	29.52 (20.39– 48.00)	28.74 (21.08– 47.70)	0.471#
	PTAL	32.83 (24.30– 45.98)	30.51 (22.10– 51.75)	29.03 (14.70–43.62)	**0.033** #
	PSAR	13.63 (5.40– 25–98)	13.69 (4.97–22.92)	11.79 (−16.00– 22.20)	0.328#
	PSAL	14.28 ± 4.57	14.94 ± 3.79	13.43 ± 4.59	0.401*
	DLNR	4.80 (1.82–8.80)	4.79 (1.61–9.00)	4.49 (1.67–12.30)	0.369#
	DLNL	4.90 (2.74–7.90)	5.01 (2.58–9.00)	5.81 (2.16–15.20)	0.662#
	DIAPR	8.74 (3.20–12.80)	8.24 (4.78–13.00)	8.39 (3.70–22.40)	0.353#
	DIAPL	9.03 (3.53–12.80)	8.28 (2.54–12.20)	8.80 (2.47–23.60)	0.396#

* Independent sample t test;

# Mann Whitney U test

## Data Availability

All data that is used in this study is available in our database.
